# Optimal evaluation of crop residues for gasification in Ghana using integrated multi-criterial decision making techniques

**DOI:** 10.1016/j.heliyon.2023.e20553

**Published:** 2023-09-29

**Authors:** Isaac Osei, Ahmad Addo, Francis Kemausuor

**Affiliations:** a Department of Renewable Energy Engineering, University of Mines and Technology, Tarkwa, Ghana; b The Brew-Hammond Energy Center, KNUST, Kumasi, Ghana; c Department of Agricultural and Biosystems Engineering, KNUST, Kumasi, Ghana

**Keywords:** Crop residues, MCDM, Gasification, VIKOR, TOPSIS, COPRAS

## Abstract

Lack of sustainable feedstock quantity and quality has been reported to be one of the major challenges confronting operations of gasifier plants in Ghana. In this paper, TOPSIS (Technique for order of Preference by Similarity to Ideal Solution) COPRAS (Complex Proportional Assessment) and VIKOR (Multi-criteria Optimisation and Compromise Solution) were used to select optimal crop residues for sustainable gasification in Ghana among thirteen residue types. The Analytical Hierarchy Process (AHP) was used as weighting criteria for the three Multi-Criteria Decision-Making (MCDM) techniques. Thirteen criteria based on particle size, proximate, ultimate analysis, calorific values, and quantity of residues were used. Five scenarios were considered; ranking of individual feedstock, consideration of the combination of the feedstock grouped under four categories. The ultimate and proximate analysis of the various crop residues as determined were generally within recommended levels as reported in the literature. Cashew shells and cassava peels have the highest heating value and annual technical residue potential of 23.4 MJ/kg and 880,750 tonnes respectively. Ranking of the individual crop residues confirmed cassava peels as the best alternatives of all the three MCDM techniques. The average rankings of the alternatives from the three MCDM in the order of best to worst are cassava peels, maize stalk, cocoa pod husk, maize cobs, rice straw, shea nut husk, groundnut husk, Palm kernel shells, EFB, rice husk, coconut husk/shells, cashew shells, and shea nut cake. Rankings of the alternatives under the four categories considered showed that feedstock blends containing four residues or more offer better opportunities for sustainable gasification than individual feedstock. Statistical analysis shows that there is a very strong, positive correlation between VIKOR, COPRAS, and TOPSIS. The strongest correlation is between VIKOR and TOPSIS with spearman's rank correlation index of 0.98. The analysis was sensitive to the weight of the strategy of group utility, recoverability ratio, residue-to-product ratio and annual crop production figures. It is recommended that optimal gasifier design and operational conditions taken into consideration the various feedstocks and their combination as determined from this study must be studied.

## 1 Introduction

Ghana has seen an increase in electricity access rate over the years, from 23.5% access rate in 1990 to 855 in 2019. However, the electricity access rate in rural areas remains lower, at about 70.5% [1]. To boost the rate of power availability in rural communities, on- and off-grid electrical options must be made available. Particularly in rural areas, renewable energy is predicted to be crucial in boosting access to energy and power. The Ghana Renewable Energy Master Plan (REMP) aims to add more bioenergy to the country's mix of energy sources [2].

The majority of fuel used in Ghana, which accounts for 37.4% of all energy consumed in the nation comes from biomass in the form of charcoal and firewood, primarily for cooking [1]. The conventional usage of biomass is unsustainable and presents associated health and environmental hazards that contribute to local and global climate change [3]. Potential substitutions for the usage of firewood and charcoal include biomass wastes from agricultural and wood-processing activities. These waste products can be used to produce heat and electricity for household and commercial uses that are sustainable and clean. There have been reports of significant annual production of biomass residues in Ghana which are currently underutilized [4]. There are therefore chances to exploit these leftovers as clean energy sources. The agriculture sector in Ghana accounts for large volumes of field and process crop residues. Aside few large-scale oil palm processing companies that utilise their waste generated for heat and electricity generation, most of the other residues are burnt openly, contributing to greenhouse gas emissions and global warming [3,4]. These residue sources can be used to generate clean energy through several conversion technologies such as torrefaction, gasification, direct combustion, pyrolysis and anaerobic digestion [3].

**Fig. 1:**
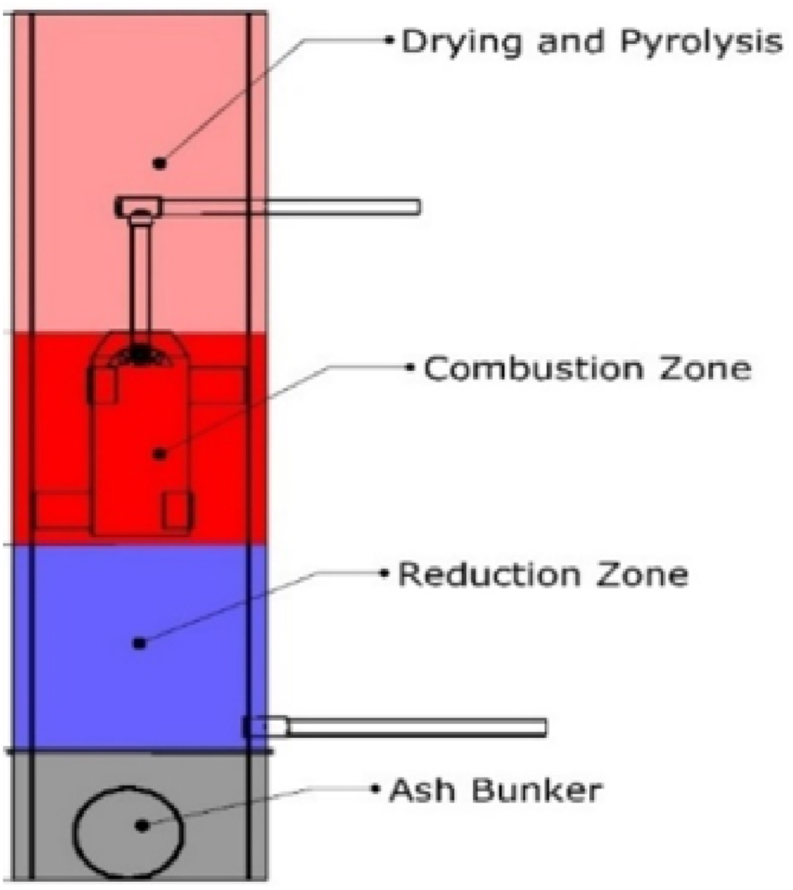
A Schematic Diagram of a Downdraft Gasifier indicating the various reaction zones.

Gasification is one of the best conversion technologies for the reuse of solid waste and it is considered as one of the most efficient ways of converting the energy embedded in biomass as it provides opportunities for small-scale applications for heat and electricity generation [3]. Gasification is the thermal treatment of biomass at higher temperatures between 600 °C to 1200 °C and in an oxygen-restricted environment which results in the generation of syngas with the constituents being Carbon monoxide (CO), Methane (CH_4_), hydrogen (H_2_), water (H_2_O), Carbon dioxide (CO_2_), heavier hydrocarbons (tars) and light (propane). The gasification process occurs in four stages and the order in which they occur depends on the gasifier reactor type (see Fig. 1). These stages include pyrolysis, drying, reduction and combustion. The biomass moisture content is reduced using heat produced from the combustion zone during the drying process in the drying zone. For gasification, biomass should have a moisture level of between 5% and 35% [5]. High biomass moisture content results in energy loss and a decline in syngas quality [6]. In the pyrolytic zone, dried biomass is thermally decomposed in the absence of air or oxygen and occurs at temperatures between 600 and 700 °C [7]. Char, gases (CO, CO_2_, H_2_, H_2_O, CH_4_), bio-oil, and tar vapours are the end-products of pyrolysis. To prevent total combustion in the combustion zone, only a portion of the fuel is oxidized using the required amount of oxygen, which is less than the stoichiometric air-fuel riaratio. The oxidation process has three key steps: partial oxidation, char combustion, and hydrogen combustion. This stage's primary output is the thermal energy needed for the entire gasification process and the combustion product, which is a combination of gases including CO_2_, CO, and H_2_O. The reduction zone of the gasification process involves the reaction of the outputs of the pyrolysis and the combustion process. The char and the gas mixture react with each other under four main reactions (boudouard reaction, water gas shift reaction, char reforming and methanation reaction) resulting in the generation of syngas. Composition, quality and quantity of syngas are dependent mostly on the gasifier type [8], gasifying medium (air, oxygen, steam or a combination) [9] operating condition (e.g., pressure, temperature, Equivalence ratio etc.) [10] and feedstock characteristics (proximate, ultimate and heating values) [9]. Syngas can be used to produce heat for cooking and drying crops. Also, when cleaned to remove CO_2_ and tar, syngas can be used in Internal Combustion Engines (ICE), fuel cells and microturbines for electricity generation. Gasification systems for crop residues have been commercially established. The reactor type has a significant impact on a gasifier's operational performance. Crop residues have been successfully used with the Inverted Down Draft (IDD) of the down-draft fixed bed gasifier type [11]. Gasifier designs are fuel-specific [11], and therefore the choice of a particular feedstock type influences the optimal gasifier design. Even though the gasification technology is quite mature and reliable, it is new in Ghana, with few installations across the country [3]. It has been reported that the main challenges confronting these facilities are inadequate and unsustainable availability of feedstock [12].

Biomass plays a critical role in the gasification process. The optimal reactor design, gasifier configuration and gasifier operating conditions (e.g., temperature, equivalence ratio, pressure etc.) are affected by the biomass type [[13], [14]]. The composition, volume, and quality of the gas produced are significantly influenced by the properties of the biomass [9]. The quantity of feedstock available also affects the sustainable operations of gasification systems [3]. The choice of fuel for optimal gasification is influenced by several factors including quantity available, fuel cost, and physical and chemical characteristics among other factors [15]. A good number of crop residues are available in Ghana which can serve as good feedstocks for gasification, however, seasonal variation of residues, as well as logistical challenges, disrupt feedstock supply which affects the smooth operation of biomass gasification operation. Co-gasification of different feedstocks can however ensure smooth operations of the gasifier. In co-gasification, two separate feedstocks are simultaneously gasified in the same gasifier. In general, it can be divided into three categories: biomass with polymer, biomass with coal, and a mixture of other biomass materials [16]. The latter combination has not been the subject of much discussion in literature, particularly for biomass types available in Ghana and many African countries [16]. The choice of feedstock or blends of feedstocks that can be used for optimal gasification in Ghana requires further investigation. A decision support system is necessary since numerous complex elements may affect the choice of optimum feedstocks for effective gasification. Multi-criteria Decision Making (MCDM) strategies can be employed in place of traditional single-criteria decision-making to tackle these complex issues.

MCDM describes any decision where multiple and conflicting criteria influence the decision Both quantitative and qualitative criteria can be handled using MCDM [17]. Multi-Attribute Decision Making (MADM) and Multi-Objective Decision Making (MODM) are the two main categories of MCDM techniques. In MADM, a number of options are compared to a set of requirements. By comparing the criteria for each alternative, the best option is chosen. In the field of renewable energy (RE), decision support systems and MCDM techniques have been employed primarily for the selection of the best RE technology and pinpointing project locations while taking into account various technical, economic, policy, and societal restrictions [18]. MCDM tools have generally been used in the bioenergy field mainly for technology and location selection [18,19], and feedstock selection [20,21]. Subjective MCDM approaches are employed when qualitative analysis is necessary, with the Analytical Hierarchy Process (AHP) being the most common [22]. Comparably, quantitative analysis uses objective MCDM methods, with TOPSIS being one of the most popular ones (Technique for Order of Preference by Similarity to Ideal Solution) [18].

One of the efficient MCDM methods for choosing the best renewable energy technology has been suggested to be Multi-criteria Optimisation and Compromise Solution (VIKOR) [18]. This method allows selections to be made as close to the optimum solution as feasible. VIKOR is an acronym for “VIseKriterijumska Optimizacija I Kompromisno Resenje” in Serbian. These methods are beneficial for both quantitative and qualitative data. They can be applied when there are several options and requirements. The compromise programming approach, which is based on an aggregating function indicating “closeness to the ideal solution,” is where TOPSIS and VIKOR techniques originate. These two techniques use various types of normalization to get rid of the criterion function units. The changes in normalization techniques could have a significant impact on the outcomes of the techniques [23]. Similar to this, the COPRAS (Complex Proportional Assessment) technique calculated the ratio of the ideal solution to the undesirable ideal solution to select the best option. COPRAS has also been identified as effective as a quantitative MCDM technique [24]. COPRAS is a fast-developed MCDM method. In this method, the influence of minimizing and maximizing criteria on the evaluation result is separately considered [25]. AHP, TOPSIS, VIKOR and COPRAS are effective tools when faced with a decision to select among several alternatives for different areas such as material selection and selection among renewable energy and particularly bioenergy technologies [17,18] and are therefore considered as the MCDM techniques in this study. Even though there have been situations where one MCDM technique has been found to be superior to another, determining whether the MCDM technique is “best” for a certain field of study is still a highly challenging task [17]. Multiple MCDMs should be employed in challenging decision situations to produce a safer and more reliable conclusion [17]. In this study, we demonstrate the application of TOPSIS, COPRAS, and VIKOR MCDM approaches for the selection of the best crop residue forof gasification in Ghana while taking into account the availability of feedstock, its physical and chemical properties, and economic factors. To verify the correctness and stability of the results, the study also includes sensitivity analysis on key decision tool factors and the choice among alternative blends of individual feedstock. Spearman's rank correlation index was used to compare the effectiveness of each of the MCDM approaches. It has been noted that integrating MCDM approaches can effectively identify the best solution [26]. The Analytical Hierarchy Process (AHP) was consequently integrated with the MCDM techniques to improve them by assigning weights to each criterion. The result for calculating weight using AHP is quite subjective, and the subjective factors may have a higher undesirable result. For this study, however, the decisions for the pairwise comparisons matrix for each criterion were based on published literature on the importance of each of the criteria to optimal gasification.

MCDM tools have generally been used in the bioenergy field mainly for bioenergy technology and feedstock selection as discussed earlier. However, none of the methods has been used for the optimal selection of crop residues for gasification. Some studies have also suggested several feedstock types for various bioenergy technologies, in most cases the selection decisions are based on one or few criteria e.g., feedstock availability, favourable chemical characteristics etc. [4,27]. However, the complexities of the factors that may influence the selection of ideal feedstocks for optimal gasification are many and therefore a decision support system is required. There are no major reported studies on the use of MCDM to select biomass feedstock for gasification, particularly for crop residues in Ghana. An integrated AHP with TOPSIS, COPRAS and VIKOR is proposed in this study. An attempt is made to select the best crop residues and their blends using feedstock characteristics, quantities available and cost as the decision criteria (thirteen criteria were considered in this study) which have not been studied. The various crop residue alternatives were selected based on unique residue types available in Ghana. The use of AHP, VIKOR, TOPSIS and COPRAS and their comparative analysis have also not been used to select residue for gasification and therefore make the approach to this study unique. The objective of this study is therefore to evaluate crop residue and their blends for optimal gasification in Ghana using VIKOR, TOPSIS, and COPRAS MCDM techniques.

The study's findings are anticipated to support the government of Ghana's efforts to create bioenergy conversion technologies by enabling the sustainable gasification of crop leftovers for on- and off-grid electrification of rural areas [2]. By presenting feedstock options and blends for the efficient operation of Ghana's existing gasification systems, the study is also anticipated to contribute to their optimal operation.

## 2 Materials and methods

**Fig. 2:**
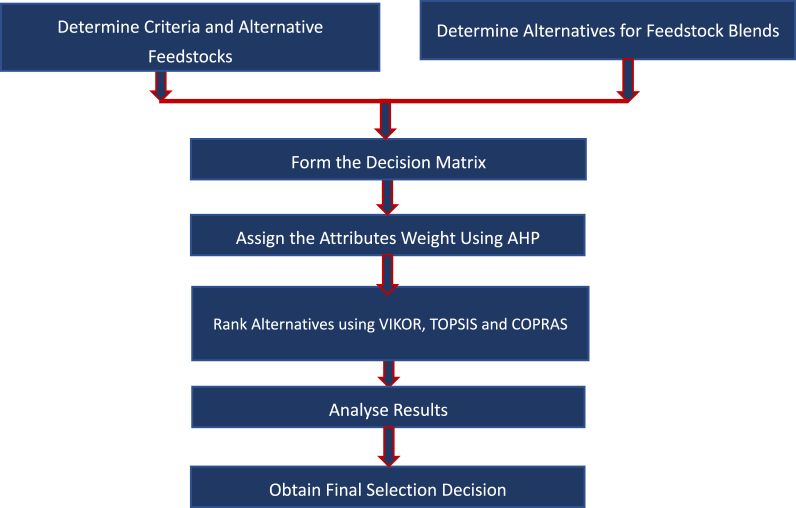
The general framework of the study.

The general framework of the study is presented in Fig. 2. A number of methods were used to achieve the overall objective of selecting an optimal crop residue and blends for sustainable gasification in Ghana.

### 2.1 Formulation of the decision-making problem

#### 2.1.1 The objective of the decision making

A number of crop residues can be used for gasification; however, these residues have different physical, and chemical characteristics, and available quantities among other factors. The objective is therefore to select the best crop residue and blends that can be used for sustainable gasification in Ghana taking into consideration feedstock availability, physical, and chemical characteristics, feedstock cost, and quantities available. According to Akolgo et al. [28], a small-scale fixed bed gasifier connected to an ICE engine generator is the type of gasification system that is now most likely to be successful in Ghana. Therefore, the goal is to select optimal feedstocks that can be used in gasifiers for efficient electricity generation in Ghana.

#### 2.1.2 Matrix of alternatives

**Fig. 3:**
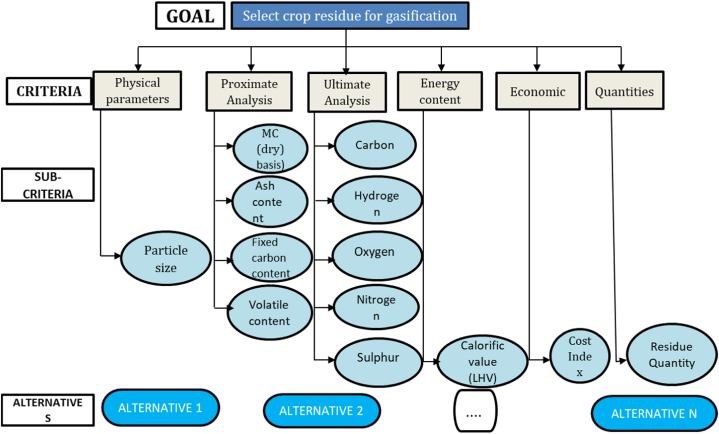
Criteria for the decision-making process.

Table 1 presents the various individual feedstock alternatives considered which were identified based on reported crop residues in Ghana that can potentially be used as feedstock for gasification [3,4,29]. Four categories were also considered for feedstock blends (see Table A1 in Appendix) based on the various group of feedstock types available across the various communities, districts, and regions in Ghana and their proximity to each other [4,30]. Crop residues in the various groups were combined to determine the various alternatives of the feedstock blends. Both individual feedstock alternatives and the alternatives for the feedstock blends were then subjected to various criteria and sub-criteria (see Fig. 3).

**Table 1:** Individual crop residue alternatives.

Symbol	Residue type (Alternatives)
A1	Rice husk
A2	Rice straw
A3	Maize stalk
A4	Maize cobs
A5	Cassava peels
A6	Cashew shells
A7	Coconut husk/shells
A8	Palm kernel shells
A9	Empty fruit bunch (EFB)
A10	Shea nut cake
A11	Groundnut shells
A12	Cocoa pod husk
A13	Shea nut shells

#### 2.1.3 List of criteria

The criteria were determined by considering critical characteristics of the feedstocks that affect optimal and sustainable gasification. These included proximate analysis (Moisture, Fixed Carbon, Ash and Volatile content), ultimate analysis (Hydrogen, Carbon, Oxygen, Nitrogen and Sulphur Content), particle size, quantities of fuel available, and costs of residues [15,31]. The reason and the role of each criterion in optimal gasification have been well discussed in subsection 3.1.2. The calorific value of feedstock has been identified as an important parameter that affects optimal gasification [32], however, there is a correlation between the elemental composition of feedstock and calorific value. Based on Dulong's formula or its modification the Carbon, Oxygen and Hydrogen content can be used to estimate the calorific value [33]. Therefore, in this study, even though the calorific value of feedstock is determined, it was not considered an independent criterion in the MCDM analysis. The individual effect of elemental Carbon, Hydrogen and Oxygen in optimal gasification is considered in this study. Table 2 presents the description of each criterion as well as the design objective for the criteria. Based on the influence of these criteria on optimal gasification, particle size, ash content, moisture content, Nitrogen and Sulphur content and cost factor were all determined to be non-beneficial attributes, therefore lower values are desirable. Percentage Volatile, Percentage Fixed Carbon, Percentage Hydrogen, Percentage Oxygen and Quantity of residues were also established as being beneficial attributes, therefore higher values are desirable.

**Table 2:** Decision criteria for the selection of the crop residues.

Criteria	Symbol	Objective	Reference
Particle size	PS	Minimise	[34]
Moisture content	MCd	Minimise	[35,36]
Ash content	PA	Minimise	[32]
Volatile content	PV	Maximise	[27,37]
Fixed Carbon content	PC	Maximise	[32]
Carbon	C	Maximise	[38]
Hydrogen	H	Maximise	[39]
Nitrogen	N	Minimise	[40]
Oxygen	O	Maximise	[35]
Sulphur	S	Minimise	[40]
Quantity of Crop residues	Q	Maximise	[31]
Cost factor	Ct	Minimise	[31]

#### 2.1.4 Methodology for feedstock characterisation

**Fig. A1:**
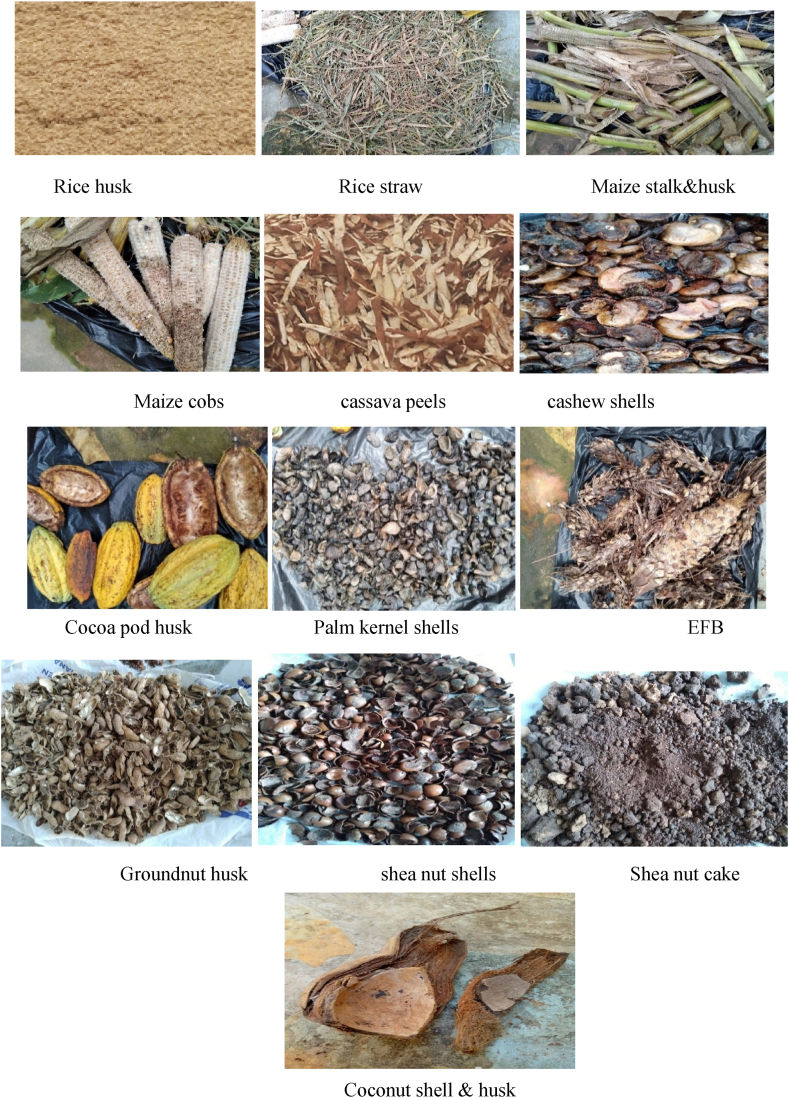
Samples of Crop Residue types used for the Proximate, Ultimate and calorific value analysis

Biomass samples were obtained from farms and agro-processing sites across Ghana during the 2021 crop production year. The samples were taken from the region in the country where the residues are produced in the largest quantities for example samples of cocoa pod husk were taken from the Ashanti region while those of shea nut husk and cake from the Northern region of Ghana. 1 kg of each sample (crop residue) was taken from three different farms or agro-processing sites. Figure A1 in the Appendix presents samples of the various crop residue types used for the study. The residues were air-dried and ground with a mechanical grinder and sieved to a fine size of less than 1 mm. The samples were labelled and stored before the proximate, ultimate analysis, and calorific value test. The calorific value (CV) of biomass feedstock was determined using a bomb calorimeter (Parr 6400 Bomb Calorimeter according to ASTM Standard E711-87). Proximate analysis (fixed carbon, volatile matter, moisture and ash content) and the ultimate analysis (Hydrogen, Carbon, Oxygen, Sulphur, and Nitrogen content) of feedstocks were performed at the Department of Chemistry and Faculty of Renewable Natural Resources, Kwame Nkrumah University of Science and Technology (KNUST), Kumasi using standards indicated in Table 3. The particle size of each sample was determined as the average diameters of the feedstock in its undisturbed state after harvesting or processing. The diameter of twenty of each feedstock type was measured and average values were determined.

**Table 3:** Standards for determining Proximate and Ultimate Analysis.

Parameter	Standard
Proximate Analysis	
Moisture content (MCd)	ASTM E 175601
Ash content (PA)	ASTM E 1755
Volatile content (PV)	ASTM E 872
Fixed Carbon content (PC)	By difference
**Ultimate Analysis**	
Carbon (C)	ASTM E 777
Hydrogen (H)	ASTM E 777
Nitrogen (N)	ASTM E 778
Oxygen (O)	By difference
Sulphur (S)	ASTM D4239

#### 2.1.5 Methodology for quantification of crop residues

The technical quantities of each of the crop residues (Q) were determined based on average annual crop production figures over a ten-year period from the year 2009–2018 [41,42]. The Residue to Product Ratio (RPR) and reported recoverability ratios in Ghana [4,43] were used to determine the dry technical residue quantities for each residue type using Equation (1) based on the methods as presented by Ref. [4]. RPR is the ratio by weight of residues to the corresponding quantity of crop (product) harvested. It is used to estimate the theoretical total amount of residues available for energy generation. However, not all residues are available as some are used for other purposes coupled with residue collection constraints. The recoverability ratio ($\eta_{reci}$) therefore indicates the realistic fraction of the theoretical residue quantities that can be assessed for energy generation. As indicated in Equation (1), the dry technical residues available annually were calculated by multiplying the average reported crop production figures by RPR, RR and the dry fraction of the feedstocks. Table A2 (see Appendix) presents the values of RPR, recoverability ratio($\eta_{reci}$), Moisture content (MC)as used in this study and the corresponding references.

(1) Q=Ci×RPRi×ηreci×((100%−MCi)100%)

Where: Q (tonnes)is the dry annual technical crop residue quantities, C_i_ (tonnes) is the average annual crop production figures from 2009 to 2018, RPR is the residue to product ratio of crop residue type i, *MC (%)* and $\eta_{rec}$ are the moisture content and recoverability fraction for crop residue type i.

#### 2.1.6 Methodology for determining cost factor (ct)

To determine the likely cost of the residues relative to each other, a residue cost factor ranging from 0 to 1 with 0 representing the lowest and 1 the highest cost was used. Factors that may affect the overall cost of the various crop residues were determined [31]. These factors included: difficulties in transportation due to low bulk density, dispersed nature of the residues, calorific values of the residues, and competition for the residues. Based on the contribution of each of these factors to the overall cost of biomass for energy generation, weights of 0.1, 0.2, 0.3, and 0.4, respectively were assigned [ [4,[44], [45], [46]]]. The weights of 0.4 for the competition of residues imply the highest competition for residues. The cost factor value for difficulties in transportation due to low bulk density [45,[47], [48], [49], [50]] and calorific values was assigned to the various feedstock types based on the relative bulk density in literature and calorific values of each residue type as determined in this study. However, the cost factor values for the dispersed nature of the residues and competition for the residues were assigned by three experts based on their experiences with these residue types in Ghana. The total cost factors for each crop residue type were then determined as the sum of individual factor values (see Table A3 in the Appendix).

#### 2.1.7 Multi-criteria decision making methods description

Three MCDM techniques were considered for this study: VIKOR, TOPSIS, and COPRAS. The AHP was used as a weighting method for each of the MCDMs.

The relative weights of the attributes (criteria) (Wi) to the objective (how each of the criteria influences the gasification process) were assigned using this method. The approach for determining AHP as stated by Cristóbal [18] was employed. However, in this instance, the relative importance of the various criteria to the target was established based on a thorough literature review on the impact each criterion has on the gasification process. The procedures for TOPSIS and COPRAS were carried out following Mousavi-Nasab and Sotoudeh-Anvari's [17] descriptions. In the case of VIKOR, the method as described by Cristóbal [18] was used.

a) VIKOR Method

This technique was created using the Lp-metric as presented in Equation (2a).

(2a) $$L_{pj}={\left\{\sum_{i=1}^{n}{\left[\frac{W_{i}\left(f_{i}^{*}-f_{ij}\right)}{\left(f_{i}^{*}-f_{i}^{-}\right)}\right]}^{p}\right\}}^{1/p}1\leq p\leq \infty ,j=1,2,\ldots .,J$$

where: n=numberofalternatives.

$W_{i}=are\;the\;weights\;of\;criteria$.

$f_{i}^{*}=”ideal”\;solution$.

$f_{i}^{-}=the\;worst\;solution$.where, L_1,j_ (as Utility Measure (S_j_) in Equation (2b)) and L_∞,j_ (as Re *gret* measure R_j_ in Equation (2c)) were used to determine the ranking measure.

(2b) $$S_{j}=\sum_{i=1}^{n}\frac{W_{i}\left(f_{i}^{*}-f_{ij}\right)}{\left(f_{i}^{*}-f_{i}^{-}\right)}$$

(2c) $$R_{j}=\max\;i\left[\frac{W_{i}\left(f_{i}^{*}-f_{ij}\right)}{\left(f_{i}^{*}-f_{i}^{-}\right)}\right]$$

where, n is the number of criteria, a_1_, a_2_,..,a_j_ indicates the J alternatives, and X_ij_ represents the ratings of the ith aspect for alternative a_j_. The following four steps were used [9].

i). *Step 1:* The decision matrix was normalized using Equation 2d

(2d) fij=Xij∑j=1nXij2wherei=1,2,….m;j=1,2,….J

i). *Step 2:* Determine $f_{i}^{-}$ and $f_{i}^{*}$ which represents the worst and best of all the criterion functions,i = 1, 2, n were determined. $f_{i}^{*}=\max\;j\;f_{ij}$ and $f_{i}^{-}=\min\;jf_{ij}$ in cases where the ith function indicates benefit. $f_{i}^{*}=\min\;j\;f_{ij}$ and $f_{i}^{-}=\max\;jf_{ij}$.

In cases where the ith function represents anything undesirable.

ii). *Step 3:* The values of S_j_ and R_j_ were then computed, j=1,2,…J by the relations as presented in Equations (2b), (2c)).
iii). *Step 4:* the value of Qj, was then computed using Equation (2e).

(2e) $$Q_{j}=\frac{v\left(S_{j}-S^{*}\right)}{\left(S^{—S^{*}}\right)}+\left(1-v\right)\left(R_{j}-R^{*}\right)\left(R—R*\right)$$

where: $S^{*}=\min_{j}S_{j}$; $S^{-}=\max_{j}S_{j}$; $R^{*}=\min_{j}R_{j}$; $R^{-}=\max_{j}R_{j}$, (1-v) is the weight of the individual regret and v is the weight for the strategy of maximum group utility. A v value of 0.5 was used in this study [17].

iv) *Step 4:* The results offer three possible ranking lists (Q, R and S) that are proposed as a compromise option, with the alternative A ^(1)^ being the best ranked by the measure Q (minimum), if the following requirements are met.
1) Acceptable advantage. $Q\left(A^{\left(2\right)}\right)-Q\left(A^{\left(1\right)}\right)\geq DQ$, where DQ=1/(J−1) and A ^(2)^ is the alternative with the second position on the ranking list by Q:
2) Acceptable stability in decision-making. Alternative A ^(1)^ must also be the best ranked by S or/and R.
3) The following compromise alternatives are suggested if one of the prerequisites in 1, 2, or both are not met:
  a. In the event that just condition 2 is unsatisfied, options A ^(1)^ and A ^(2)^ should be selected or
  b. Alternatives A ^(1)^, A ^(2)^, …., A ^(M)^ if condition 1 is not satisfied.

Where A ^(M)^ is determined by the relation Q (A ^(M)^) – Q (A ^(1)^) < DQ for the maximum n.

b) TOPSIS

The following four steps were used to rank the various alternatives.

i). *Step 1:* Equation (3a) was used to normalize the decision matrix.

(3a) rij=Xij∑j=1nXij2wherei=1,2,….,m;j=1,2,…,n

ii) *Step 2:* The matrix was weighted using Equation (3b).

(3b) $$V_{ij}=w_{j}\times r_{ij}\;where\;i=1,2,\ldots .,m;j=1,2,\ldots ,n$$

$$w_{j}=is\;the\;weight\;of\;the\;criteria\;as\;determined\;from\;the\;AHP$$

*iii). Step 3:* The nadir solution ($A^{-})$ and the best Ideal Solution $\left(A^{*}\right)$ were defined as follows:

$$A^{*}=\left\{V_{1}^{*},V_{2}^{*},\ldots ..,V_{n}^{*}\right\}=\left\{\left(\max_{j}v_{ij}|i\in I^{\prime }\right),\left(\min_{j}v_{ij}|i\in I^{\prime \prime }\right)\right\}$$

i=1,2,…..,m;j=1,2,…,n.

$$A^{-}=\left\{V_{1}^{-},V_{2}^{-},\ldots ..,V_{n}^{-}\right\}=\left\{\left(\min_{j}v_{ij}|i\in I^{\prime }\right),\left(\max_{j}v_{ij}|i\in I^{\prime \prime }\right)\right\}$$

i=1,2,…..,m;j=1,2,…,n.

where, I′ is tied to beneficial traits, while I″ is associated with attributes that are non-beneficial.

iv) *Step 4:* Equations (3c), (3d)) were used to achieve the remoteness of all choices from $A^{+}$ and $A^{-}$.

(3c) $$D_{i}^{+}=\sqrt{\sum_{j=1}^{n}{\left(v_{ij}-v_{j}^{+}\right)}^{2}}\;i=1,2,\ldots ..,m$$

(3d) $$D_{i}^{-}=\sqrt{\sum_{j=1}^{n}{\left(v_{ij}-v_{j}^{-}\right)}^{2}}\;i=1,2,\ldots .,m$$

v). *Step 5*: Equation (3e) was used to determine the relative closeness to the perfect solution.

(3e) CCi*=Di−Di−+Di+i=1,2,….,m

vi) *Step 6:* The alternatives were then prioritised using ${CC}_{i}^{*}$. The larger ${CC}_{i}^{*}$ indicates better accomplishment of options.
c) COPRAS

COPRAS was used to rank the alternatives using the following steps:

i). *Step 1:* The decision matrix was normalized using Equation (4a).

(4a) rij=Xij∑i=1mXij

ii) *Step 2:* Equation (4b) was used to weight the matrix $\left(r_{ij}\right)$.

(4b) $$y_{ij}=w_{j}\times r_{ij}\;\left(i=1,2,\ldots .,m;j=1,2,\ldots ,n\right)$$

iii) *Step 3:* Equations (4c), (4d)) were then used to obtain the sums of the weighted normalized scores for the cost and benefit criterion respectively.

(4c) $$S_{+i}=\sum_{j=1}^{n}y_{+ij}$$

(4d) $$S_{-i}=\sum_{j=1}^{n}y_{-ij}$$

$y_{+ij}$ is associated with maximizing criterion, whereas $y_{-ij}$ is related to minimizing criteria as presented in Equations (4c), (4d)).

iv) *Step 4:* The relative priority of the alternatives was then obtained using Equation (4e). The alternative with the highest value of $Q_{i}\left(Q_{\max}\right)$ is the best.

(4e) $$Q_{i}=S_{+i}+\frac{\min\left(S_{-i}\right)\sum_{i=1}^{m}S_{-i}}{S_{-i}\sum_{i=1}^{m}\frac{\min\left(S_{-i}\right)}{S_{-i}}}$$

v). *Step 5:* The level of performance ($U_{i})$ for the option I was computed using Equation (4f).

(4f) Ui=QiQmax×100

where: $Q_{\max}$ is the highest relative importance score.

#### 2.1.8 Methodology for comparing the MCDM methods using SRCI

Spearman's Rank Correlation Index (SRCI) was used to analyse the statistical significance of resemblance between different pairs of rankings. The SRCI ranges from −1 to +1, with a value of +1 denoting perfect agreement between ranks and a value of −1 denoting perfect disagreement between ranks. Equation (5) was utilized to calculate the SCRI.

(5) rs=1−6∑d2n(n2−1)

Where: n is the number of alternatives, $r_{s}$ is Spearman's correlation index and d is the difference between the rankings of the alternatives. The following verbal descriptions of the correlation strength $(r_{s}$) between the approaches used: 0.00–0.19 ″very weak,” 0.20–0.39 ″weak,” 0.40–0.59 ″moderate,” 0.60–0.79 ″strong,” and 0.80–1.0 ″very strong."

To determine whether there is proof that a linear connection exists in the population, a significance test was run. To do this, we test the alternative hypothesis, H_1_, that there is a monotonic correlation, against the null hypothesis, H_0_, that there is no correlation (monotonic correlation) in the population

$$H_{0}:\rho_{s}=0$$

$$H_{1}:\rho_{s}\neq 0$$

where ***ρ***_s_ is the Spearman's population correlation coefficient.

A two-tailed test was conducted with a significance level (α value of 0.05) to determine the kind of correlation between the decision-ranking techniques. Whenever the probability distribution p−value is less than α=0.025 [51], We reject the null hypothesis since it shows that there is a statistically significant association between the obtained ranks of the two compared approaches. The p values were determined by statistical test using an inbuilt Microsoft Excel formula as presented in Equation (2a), (2b), (2c), (2d), (2e) The p values were subsequently determined using an inbuilt Microsoft Excel function as presented in Equation (6).

(6) p=TDIST(t,Df,2)

where: TDIST is an inbuilt Excel function that calculates the p-value when t, Df and the type of tail test are supplied as input, t is the statistical test value (see Equation (7)), Df is the degree of freedom and was determined as n-2 where n is the total sample size (i.e. alternatives). Finally, 2, represents the type of tail test which is a two-tail test for this analysis.

(7) $$t=\frac{r_{s}}{\sqrt{\left(1-r_{s}^{2}\right)/\left(n-2\right)}}$$

where t is the test statistics, $r_{s}$ is the Spearman rank correlation index between the two compared MCDM techniques and n is the sample size (i.e. alternatives).

#### 2.1.9 Sensitivity analysis

The critical variables within each of the MCDM methods were subjected to sensitivity analysis. The weight for the strategy of maximum group utility (v) in the VIKOR method, residue to product ratio and lower and upper bounds values quantity of each feedstock were subjected to the sensitivity analysis.

## 3 Results and discussions

### 3.1 Pairwise comparison matrix for the criteria

A pairwise comparison matrix using a scale of relative importance was constructed (see Table A4 in the Appendix). The relative importance or otherwise of each criterion compared to each other was based on published literature on how each contributes to optimal and sustainable gasification [16,32,35,40]. A consistency concern arises when performing pairwise comparisons in AHP particularly when the number of criteria is beyond three. A consistency ratio (CR) was therefore determined to ascertain the consistency in the judgements made. A consistency ratio of less than one is desirable. In this study, a CR ratio of 0.097 was determined indicating that, the pairwise comparison matrix is consistent and there is a good consistency in the decision made [18].

### 3.2 Determination of weights of relative importance (Wi) using AHP

**Fig. 4:**
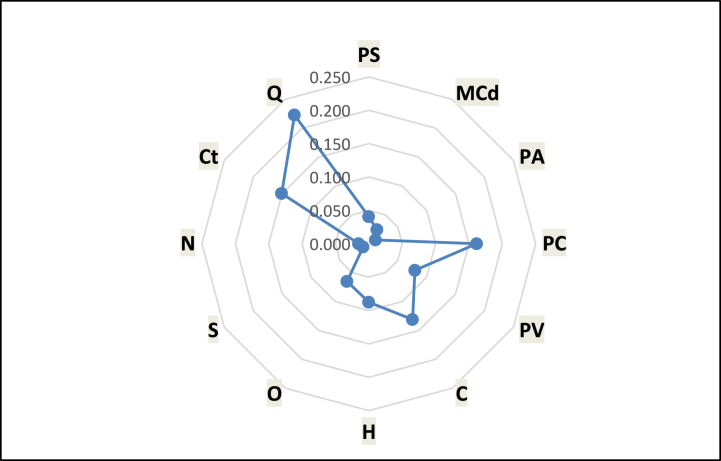
Weights of criteria.

In the AHP the relative importance of each of the criteria is determined. The criterion with the biggest weight is the most important factor to consider for optimal gasification. The results of the research showed that the quantity of feedstock readily available, with a weight of 0.22, is the most important criterion to consider in optimal and sustainable gasification. Fig. 4 shows the weight of each of the criteria. This outcome is in line with the literature on the critical role biomass availability plays in the uptake, optimal and sustainable operations of gasification systems [14,28]. The lack of sustainable feedstock supply has contributed to the collapse of gasifier plants in Ghana [3]. The disruption of biomass supply could prevent the exploitation of the technology for an extended moment. Sulphur content had the least weight of 0.009. This is because sulphur-containing compounds in biomass transform to sulphur dioxide (SO_2_) in an oxidising atmosphere and hydrogen sulphide (H_2_S) in a reducing atmosphere during gasification. The presence of these gaseous sulphur compounds raises both operational and environmental concerns. They cause corrosion in downstream processing requiring the need for effective desulphurisation which results in increased overall investment and operational cost of gasification projects. They contribute to acid rain when released into the atmosphere. The various weight of the other criteria as presented in Fig. 4 corresponds to the role each play in the optimal gasification process. This has been extensively discussed in sub-section 3.3.

### 3.3 Decision criteria values for the alternatives

The thirteen feedstock alternatives and the corresponding criteria are presented in Table 4. Proximate and ultimate analysis plays a critical role in determining, the quantity and quality of syngas from gasification and affects gasifier design and operation [15]. The results were mostly within the ranges reported in the literature [36,40]. In some cases, the values determined varied slightly from the reported cases literature due to differences in crop variety, climatic conditions, and soil composition, among other factors [51].

**Table 4:** Alternatives and criteria.

Alternatives	**PS** (mm)	MCd (wt.%)	PA (wt.%)	PC (wt.%)	PV (wt.%)	C (wt.%)	H (wt.%)	O (wt.%)	**S** (wt.%)	N (wt.%)	CV (MJ/kg)	Ct	Q (t)10^3^
A1	2.67 ± 0.24	7.15 ± 0.46	20 ± 0.26	12.8 ± 0.61	59.97 ± 2.00	40.1 ± 0.59	5.2 ± 0.08	34 ± 1.53	0.7 ± 0.03	0.16 ± 0.02	12.9 ± 0.33	0.32	93.17
A2	2.10 ± 0.11	8.68 ± 0.19	9.46 ± 0.85	9.76 ± 0.90	72.11 ± 3.52	39.9 ± 1.07	5.9 ± 0.15	43 ± 1.81	0.6 ± 0.03	0.85 ± 0.08	14.6 ± 0.25	0.49	525.09
A3	14.80 ± 0.83	8.27 ± 0.32	7.92 ± 0.30	12.8 ± 0.42	71.01 ± 0.77	44.1 ± 0.59	6.5 ± 0.12	40 ± 0.59	0.4 ± 0.03	0.99 ± 0.03	14.3 ± 0.22	0.46	749.33
A4	22.50 ± 3.82	9.05 ± 1.03	2.21 ± 0.18	13.0 ± 0.58	75.72 ± 0.64	43.5 ± 0.87	6.9 ± 0.06	47 ± 0.27	0.2 ± 0.01	0.64 ± 0.06	15.7 ± 0.19	0.49	338.33
A5	13.89 ± 0.78	7.52 ± 0.59	4.25 ± 0.11	10.1 ± 0.15	78.10 ± 1.08	45.1 ± 1.05	6.2 ± 0.16	42 ± 0.29	0.1 ± 0.03	2.21 ± 0.43	14.6 ± 0.21	0.55	880.75
A6	26.60 ± 2.55	6.90 ± 0.01	1.79 ± 0.09	20.9 ± 0.85	70.39 ± 1.15	52.7 ± 0.62	7.1 ± 0.10	38 ± 0.70	0.1 ± 0.01	0.71 ± 0.02	23.4 ± 0.15	0.82	10.18
A7	94.17 ± 5.68	8.04 ± 0.32	1.51 ± 0.21	16 ± 0.75	74.40 ± 0.83	50.3 ± 1.25	5.3 ± 0.26	42 ± 0.50	0.1 ± 0.01	0.50 ± 0.03	16.5 ± 0.05	0.53	135.13
A8	17.78 ± 1.26	8.23 ± 0.15	1.91 ± 0.37	20.3 ± 0.39	69.57 ± 1.66	45.5 ± 0.29	5.6 ± 0.19	46 ± 0.10	0.3 ± 0.03	0.57 ± 0.02	18.5 ± 0.08	0.58	113.68
A9	25.88 ± 1.74	7.45 ± 0.33	5.77 ± 2.14	13.1 ± 0.38	73.71 ± 0.84	44.5 ± 1.26	6.7 ± 0.07	42 ± 0.58	0.5 ± 0.06	0.78 ± 0.05	14.6 ± 0.22	0.50	171.18
A10	1.50 ± 0.21	6.47 ± 0.84	7.78 ± 1.23	14.5 ± 0.29	71.25 ± 0.78	46.9 ± 1.56	5.5 ± 0.12	37 ± 0.29	0.6 ± 0.01	2.06 ± 0.05	22.9 ± 0.09	0.60	3.21
A11	19.20 ± 2.32	7.76 ± 0.83	3.67 ± 0.36	20.3 ± 0.81	68.27 ± 0.51	48.7 ± 1.23	6.4 ± 0.21	40 ± 0.10	0.3 ± 0.01	0.78 ± 0.04	16.2 ± 0.21	0.49	110.75
A12	92.50 ± 4.37	10.98 ± 0.32	7.86 ± 0.35	16.6 ± 0.25	64.57 ± 0.53	42.1 ± 0.59	5.7 ± 0.10	42 ± 0.27	0.8 ± 0.01	1.49 ± 0.11	14.3 ± 0.12	0.31	555.89
A13	19.70 ± 1.87	3.56 ± 0.72	1.84 ± 0.14	22.7 ± 0.24	71.95 ± 0.86	50.9 ± 1.07	6.7 ± 0.26	40 ± 0.58	0.2 ± 0.01	0.32 ± 0.01	17.3 ± 0.19	0.43	10.15

The proximate and ultimate analyses are on an air-dry basis.

Higher moisture content in gasification promotes poor ignition [36]. Fuels with low moisture produce more carbon monoxide (CO) [35]. Cocoa pod husk and shea nut husk had the highest and lowest moisture content of 10.98% and 3.56% respectively. The thermal decomposition and combustion behaviour of fuels are significantly impacted by the high amount of volatile matter [37].

At temperatures below 600 °C, the volatile part of the fuel is vaporized during gasification. Tar, carbon monoxide, hydrocarbon gases, carbon dioxide, hydrogen water vapour, and carbon dioxide make up the volatile vapours. The gases are required in the reduction zone to produce hydrogen, methane, and carbon monoxide. For example, CO_2_ is required to produce CO through the boudouard reaction which is a desirable end product of gasification. However, high volatile matter also produces significant levels of tar, a gasification byproduct that should be avoided [35]. However, by using the best gasifier operating settings, such as the equivalency ratio, temperature, pressure, and or the use of a catalyst, the tar content can be decreased. High volatile content is therefore often a desirable property of fuels in the gasification process. The cost of handling and processing biomass has an impact on conversion technology costs. High ash content poses operating issues for gasifiers that could result from slagging because of the development of clinkers. Therefore, low ash content in feedstocks is preferred for optimal gasification [32]. Cashew shells, coconut husk/shells, shea nut shells, and palm kernel shells presented lower values compared to values in the literature. Because the carbon remaining after devolatilization is used for gasification in the reduction reaction zone, a larger fixed carbon content is preferred in gasification processes [31]. Shea nut shells had the highest fixed carbon content of 22.7%.

The quality and composition of syngas are affected by the elemental composition of the feedstock. Higher elemental carbon is required in the gasification reaction because higher values increase the carbon available for the gasification process which is required in all the four major chemical reactions in the reduction reaction zone to produce, H_2_, CH_4,_ and CO [38]. High oxygen content in feedstocks Implies that less oxygen as an oxidant is required during the gasification process [35]. However, the amount of carbon in the biomass fuel is constrained by high oxygen levels. The amount of sulphur and nitrogen oxides in the syngas increases due to greater elemental nitrogen and sulphur content in the biomass feedstock. These constituents of the syngas are undesirable and therefore lower elemental values of Nitorgrn and Sulphur are desirable in the gasification reaction. The sulphur contents were within the ranges reported in the literature. However, higher sulphur content was observed in rice husk, rice straw, shea nut cake, and cocoa pod husk and this may be a result of differences in crop variety type considered, climatic conditions and soil composition [52]. The calorific values are within the ranges reported in the literature for the feedstocks under consideration. A higher calorific value in the feedstock is desirable for gasification as it contributes to the heating value of the syngas. Cashew shells and rice husks had the highest and least calorific values of 23.4 MJ/kg and 12.9 MJ/kg respectively. In this study, the calorific value served as an input parameter in the determination of the various cost factors of the crop residues considered as discussed in section 2.1.6. The particle size, defined by the average diameters of a feedstock in its undisturbed state after harvesting or processing, was determined. The quality of syngas composition has been reported to increase with a decrease in particle size [34]. Feedstocks with larger particle sizes must undergo size reduction before usage which may increase overall investment and operational cost of energy generation. The quantity of feedstocks plays a critical role in optimal syngas generation. Most studies in Ghana use crop production figures of one year for residue estimation and in some cases RPR and recoverability ratios from literature outside Ghana [3]. The estimation in this study, however, used average crop production figures for ten years and RPR and recoverability ratios from literature determined from fieldwork in Ghana. Table A2 in the Appendix presents the estimation of the various crop residues types, corresponding crop production figures, RPR and recoverability ratios. Based on the crop residue types considered, a total of 3.69 Mt of crop residues are generated in Ghana annually. Kemausuor et al. [4] estimated, total annual quantities of crop residues of 18.86 Mt with twenty-nine different residue types considered compared to the thirteen residue types considered in this study. Cassava peels had the highest technical residue quantity of 880,750 tonnes/y (see Table 4). Kemausuor et al. [4] established cassava peels as one of the residues with the highest technical residue potential in Ghana. Their study established annual technical residue quantities of cassava peels of 977,060 tonnes, higher than the estimated value (880,750 tonnes) in this study. The difference is accounted for by the different crop production figures used. Whiles average crop production figures for ten years were used in this study, Kemausuor et al. [4] used the crop production figures for a single year (2011). The outcomes of the study indicate that significant quantities of crop residues particularly cassava peels, maize stalk and husk, cocoa pod husk, rice straw and maize cobs are available in Ghana and can be used for optimal gasification.

### 3.4 Ranking of alternatives by VIKOR, TOPSIS and COPRAS for individual feedstocks

**Fig. 5:**
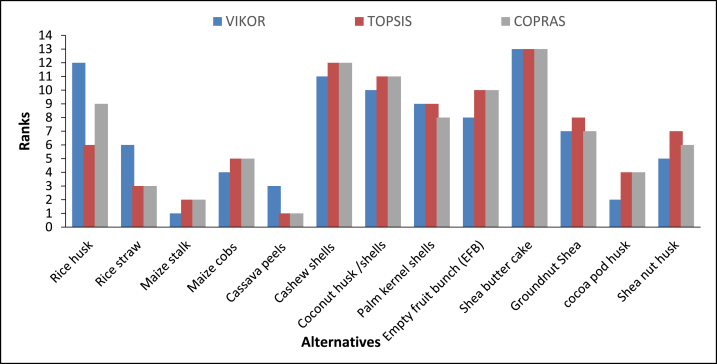
Ranking of alternatives using VIKOR, TOPSIS, and COPRAS.

**Fig. 6:**
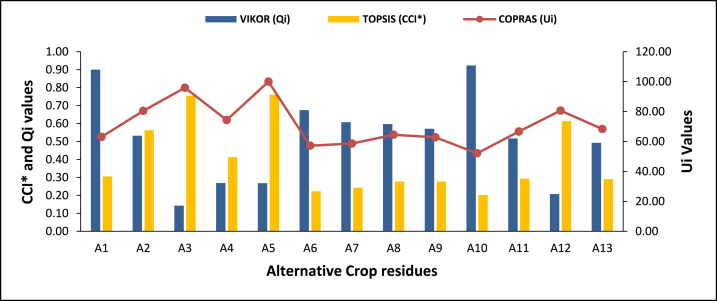
Qi, Ui, and CCI* values for the alternatives.

Based on the weights of relative importance (Wi) and the decision criteria, the various alternatives were ranked by the three MCDM techniques. Cassava peel (A5) was ranked as the best crop residue for optimal and sustainable gasification in Ghana by TOPSIS and COPRAS. However, VIKOR ranked maize stalk as the best feedstock for optimal gasification in Ghana (see Fig. 5). Fig. 6 presents the various parameters used to rank the alternatives. In the VIKOR method Condition ‘a’ i.e., was not satisfied and therefore a compromise solution is made between A3 (maize stalk) ranked first and A12 (cocoa pod husk) ranked second. The outcome of the analysis confirms Awafo and Agyemang [29] report that cassava peels are one of the best feedstocks for energy generation in Ghana. Cocoa pod husk was determined to be one of the best crop residues for optimal gasification in Ghana. VIKOR ranked cocoa pod husk as the second-best feedstock while both TOPSIS and COPRAS ranked it as the third-best feedstock. Contrary to this finding, Nelson et al. [27] identified cocoa pod husk as the most important biomass resource because there are currently not many competing uses. However, this may not be the best feedstock for gasification in Ghana just based on residue availability. The multiplicity of factors that may affect the use of the residue for gasification must be taken into consideration and compared with other potential available feedstocks to identify the optimal residue as done in this study. The average ranking of the alternatives from the three MCDM in the order of best to worst alternatives are; cassava peels, maize stalk, cocoa pod husk, maize cobs, rice straw, shea nut husk, groundnut husk, Palm kernel shells, EFB, rice husk, coconut husk/shells, cashew shells, and shea nut cake.

The similarity and dissimilarity of the final alternative rankings generated by the various decision-ranking strategies were assessed using the Spearman's rank correlation (see Table 5). There is a statistically significant association between the rankings of the crop residues by the three MCDM procedures since the p-value in each case is less than −0.025, which leads us to reject the null hypothesis. There was a very strong, positive correlation between TOPSIS and COPRAS with a correlation index of 0.962. Among the three MCDM techniques, the correlation between VIKOR and TOPSIS was the lowest with an index of 0.813. Some factors such as the weight for the strategy of maximum group utility (v), recoverability ratio, residue-to-product ratio, and crop production figures were determined to be sensitive to the outcomes of the simulations.

**Table 5:** Spearman rank correlation and statistical test between MCDM Techniques for the various scenarios.

Scenarios	MCDM Technique	Spearman correlation (rs)	N	T statistics	Degree of freedom (DF)	Sig. (2-tailed)
Individual feedstocks	VIKOR & TOPSIS	0.813	13	4.63	11	0.00072
	VIKOR & COPRAS	0.906	13	7.16	11	1.927E-05
	TOPSIS & COPRAS	0.962	13	11.61	11	1.632E-07
Category Ca	VIKOR & TOPSIS	0.975	255	69.288	253	1.627E-166
	VIKOR & COPRAS	0.867	255	27.64	253	2.207E-78
	TOPSIS & COPRAS	0.911	255	35.04	253	4.770E-99
Category Cb	VIKOR & TOPSIS	0.981	255	69.28	253	1.627E-166
	VIKOR & COPRAS	0.863	255	27.64	253	2.207E-78
	TOPSIS & COPRAS	0.911	255	35.04	253	4.770E-99
Category Cc	VIKOR & TOPSIS	0.970	31	21.40	29	2.61383E-19
	VIKOR & COPRAS	0.915	31	12.24	29	5.59614E-13
	TOPSIS & COPRAS	0.948	31	16.04	29	5.91044E-16
Category Cd	VIKOR & TOPSIS	0.966	31	20.04	29	1.56403E-18
	VIKOR & COPRAS	0.904	31	11.33	29	3.58273E-12
	TOPSIS & COPRAS	0.9474	31	15.83	29	8.18454E-16

### 3.5 Ranking of alternatives by VIKOR, TOPSIS and COPRAS for categories Ca, Cb, Cc, and Cd

**Fig. 7:**
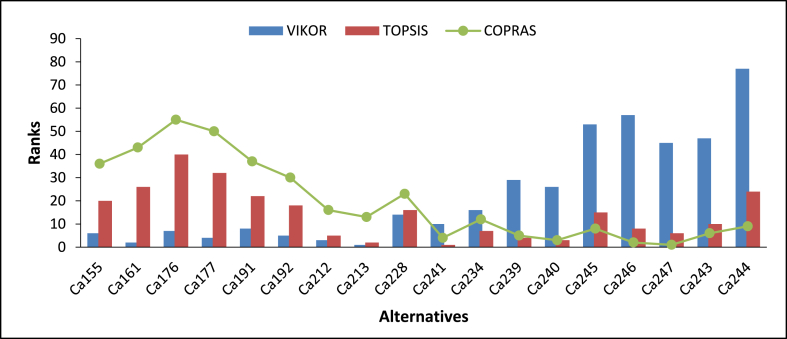
Ranking of alternatives for category Ca, using VIKOR, TOPSIS, and COPRAS.

**Fig. 8:**
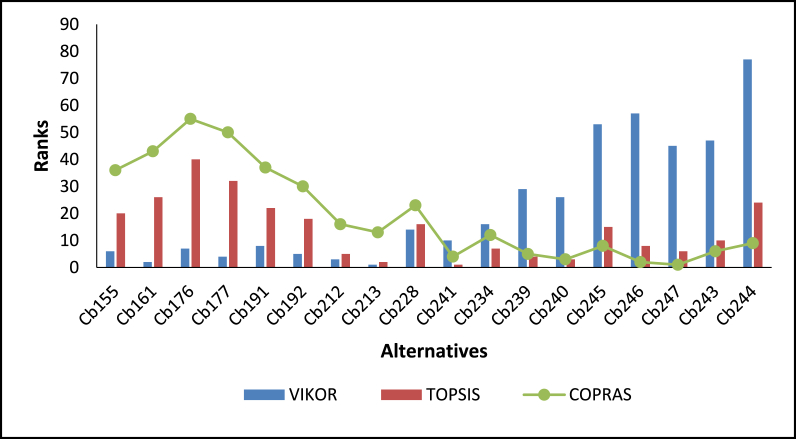
Ranking of alternatives for category Cb, using VIKOR, TOPSIS, and COPRAS.

**Fig. 9:**
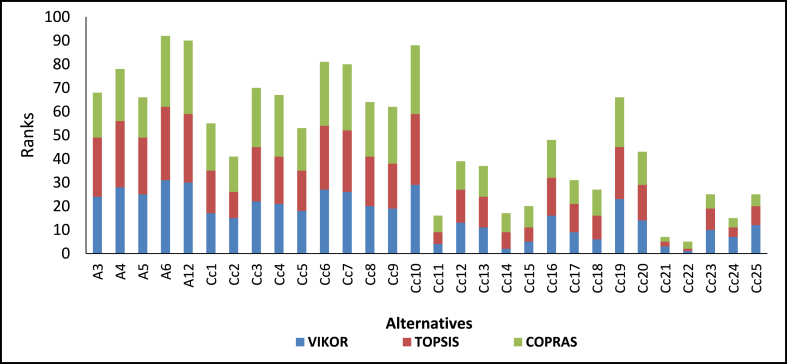
Ranking of alternatives for category Cc, using VIKOR, TOPSIS, and COPRAS.

**Fig. 10:**
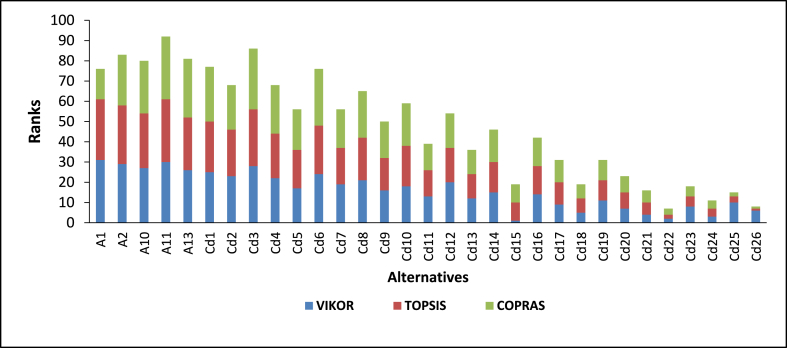
Ranking of alternatives for category Cd, using VIKOR, TOPSIS, and COPRAS.

The alternatives of the various feedstock blends under the four categories were ranked. In the case of feedstocks under categories Ca and Cb, 255 alternatives were considered, while for Cc and Cd, 31 alternatives consisting of individual feedstocks and their blends were considered. Generally, the results of the various categories show that alternatives with a higher number of feedstocks are better than alternatives with less (see Fig. 7, Fig. 8, Fig. 9, Fig. 10).

The details of the various rankings of the feedstocks under each category are presented in paragraphs 2–6 of this section. In all the categories considered, feedstock blends were ranked ahead of individual feedstock with an increasing number of feedstocks in the blends preferred. For categories Ca and Cb, the alternatives ranked among the first ten had at least five feedstock blends. This indicates that for optimal and sustainable gasification, co-gasification considering available feedstock blends has better prospects. Similar findings have been reported [16]. The use of various feedstock blends harnessed the advantages of each of the feedstock types. For instance, some feedstock may have very good chemical characteristics for optimal gasification but the quantities available may not be enough e.g. cashew shells, on the other hand, some categories of feedstock types may not have favourable chemical properties but have enough quantities available e.g. rice straw. Additionally, feedstock mixes can present a variety of options, such as the addition of scarce individual fuels to get an adequate quantity, as in the case of seasonally available biomass fuels, which are not always available. This finding is important, particularly in the case of Ghana where sufficient quantities of one feedstock type may not be available, however, due to the farming system different crop residue types may be available within a particular location. Akolgo et al. [28] reported that for a gasifier plant to be successful in Ghana it should be able to use a wide range of feedstock types and properties. Considering feedstock blends can therefore solve problems with some installed gasifier plants in Ghana. One of the major problems for the 120 kWe downdraft gasifier plant at Asueyi, in the Brong-Ahafo area of Ghana, was reported to be inconsistent feedstock availability [12]. The facility makes use of cassava peels generated in the agro-processing industry. Other available feedstocks such as rice husk maize cobs, cashew shells etc. [3], if exploited could have ensured the optimal and sustainable operation of the gasifier plant. Interestingly, it is not all cases where alternatives with a higher number of feedstock blends were ranked ahead of the ones with lower numbers. For example, Alternative Ca161, consisting of five feedstock blends, was ranked second for VIKOR ahead of alternatives with eight feedstock blends. Similar observations were made for the rankings by TOPSIS and COPRAS. This outcome, therefore, indicates that the individual characteristics of each feedstock type within the blends play a critical role.

Alternatives with at least a rank within the first ten for any of the three techniques were considered in the graph for category Ca. The same approach was used for category Cb. Under category Ca (see Table A5, Table A6 in the Appendix for the feedstocks that constitute the various alternatives in Ca and Cc respectively) different best alternatives were ranked by each of the three techniques. Ca213, Ca241, and Ca247 were ranked as the best alternatives by VIKOR, TOPSIS, and COPRAS respectively. In the VIKOR method, condition ‘a’ was not satisfied and therefore a compromise solution is made between Ca213 ranked first and Ca161 ranked second. There was a very strong, positive correlation between VIKOR and TOPSIS (rs = 0.975, n = 255, p < 0.001). A similar relationship existed between the other two combinations. On average, Ca241 consisting of seven residue combinations was ranked as the best alternative by the three techniques.

In category Cb, three different alternatives were ranked as the best by the three techniques. VIKOR, TOPSIS, and COPRAS identified alternatives Cb213, Cb241, and Cb247 respectively as the best. Based on the average ranking of the three MCDM techniques Cb213 was ranked as the best. This consists of six feedstock types comprising of, rice husk and straw, maize stalk and cob, cassava peels and cocoa pod husk. Despite the different rankings by three MCDM techniques, statistical analysis shows that there is a very strong positive correlation between them. The strongest positive correlation is between VIKOR and TOPSIS (rs = 0.981, n = 255, p < 0.001). This is contrary to the statistical analysis presented in the individual feedstock analysis where the strongest correlation was between TOPSIS and COPRAS. This seems to suggest that, as the number of alternatives increases the correlation between VIKOR and TOPSIS becomes stronger. Similar findings have been reported with Spearman's rank correlation index of 0.946 between VIKOR and TOPSIS for 176 alternatives [53]. The strength of the other correlation also increased with the number of alternatives (see Table 5). One of the envisaged challenges of dealing with energy generation involving a number of feedstocks is the ability to get access to them within a considerable radius. However, based on Ghana's mixed cropping system, these residues can be found in clustered small-scale, irrigated farms and agro-processing centres [54]. Under Category Cc, Cc22 was ranked as the best alternative by VIKOR and TOPSIS, with COPRAS ranking it as the third best. This category consists of maize stalks and cobs, cassava peels and cocoa pod husks. COPRAS ranked C26 consisting of five feedstocks as the best alternative. This feedstock blend consist of maize stalk and cobs, cassava peels, EFB, Cashew shells, and cocoa pod husk. Statistical analysis shows that there is a very strong, positive correlation between the three MCDM techniques.

**Fig. 11:**
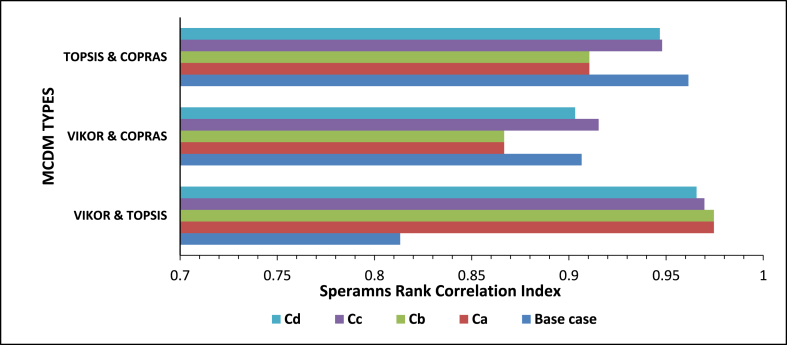
Comparison of correlation significance of VIKOR, TOPSIS and COPRAS.

Thirty-one alternatives were considered in Category Cd. Contrary to the ranking pattern presented in Cc, the alternative with the highest number of feedstock (Cd26) was ranked as the best alternative by both TOPSIS and COPRAS. This consists of five individual feedstocks (rice husk and straw, shea nut cake and shells and groundnut shells). VIKOR on the other hand ranked Cd15 as the best alternative which consists of all the feedstocks presented in Cd26 except rice husk. The Spearman rank correlation between the techniques shows that there is a very strong, positive correlation between the three MCDM techniques in the ranking of the feedstock blends in this category (see Table 5). Similar to the results presented in the other categories, it can be seen that alternatives with a higher number of feedstocks are ranked ahead of those with fewer or individual feedstocks. Moreover, the statistically significant correlations between MCDM techniques also increase with an increase in the number of alternatives (see Table 5). Zamani-Sabzi et al. [51] reported similar outcomes. The Spearman rank correlation varies even in situations where the number of alternatives is the same as shown in Ca and Cb; Cc and Cd (see Table 5). As the number of alternatives increases, the correlations between TOPSIS and VIKOR increase (see Fig. 11). On the contrary opposite effect on the size of alternatives on the spearman's rank correlation was observed between TOPSIS and COPRAS as well as VIKOR and COPRAS (see Fig. 11).

### 3.6 Sensitivity analysis of the MCDM techniques

The base case scenarios consisting of the ranking of individual crop residues and the various categories were subjected to sensitivity analysis to identify critical parameters that may affect the decision outcomes and how the variation of such parameters affects the final ranking of the alternatives. The RPR, crop production figures, Recoverability Ratio, and weight of the strategy of group utility were identified to be sensitive to the MCDM techniques.

**Fig. 12:**
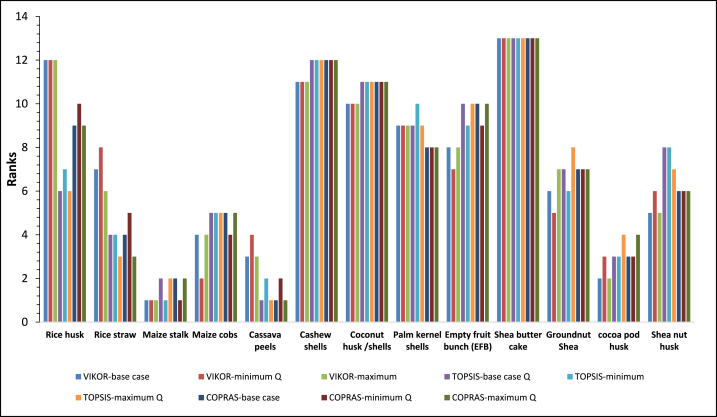
**Effects of variation of feedstock quantity on the rankings of optimal alternative by** VIKOR, TOPSIS and COPRAS.

The quantity of the residues played a critical role in the rankings of the alternatives by the three MCDM techniques as observed in section 3.4. The quantities of residue available are directly affected by the crop production figures, RPR and recoverability ratios. An average crop production figure within ten years was used in the base case study to estimate the quantity of feedstock available for each residue type. Based on the least and highest annual crop production figures (see Table A2 in Appendix), the corresponding lower and upper limits of the dry technical residues (Q) were used to rank the various alternatives. Based on the lowest dry technical residues that can be available for each feedstock, maize stalk was ranked as the optimal crop residue. Based on the upper limit value of Q, cassava peels were ranked as the optimal crop residue. It was observed, that, the best three residues in the base case were ranked among the first three residue types in situations where lower or upper bound values of Q are used to rank the alternatives. The effects of the lower bound and upper bound values of Q on the ranking of each residue varied differently (see Fig. 12). The rankings of cashew shells, coconut husk/shells and shea butter cake showed minimal variation (see Fig. 12). This is the result of the minimal variability in the annual crop production data for these feedstocks. Similar effects of lower and upper bound values of Q on the alternative rankings were observed for all feedstock categories. The recoverability ratio is the fraction of the crop residue types that can be collected for energy generation taking into consideration constraints in residue collection and utilisation of the residues among others. The recoverability ratio plays a crucial role in the estimation of the quantity of crop residues available for energy generation [55]. Groode and Heywood [56] suggested a recoverability ratio for crop residues of 0.3–0.5. A recoverability ratio of 0.2 was used for cassava peels (in the base case scenario) which were ranked as the best alternatives for the individual feedstock rankings. Sensitivity analysis revealed RR of cassava peels below resulted in the ranking of maize stalk as the optimal crop residue by all three MCDM techniques. Similarly, VIKOR ranked maize stalk as the optimal crop residue in the base case scenario with a recoverability ratio of 0.35. However, the recoverability ratio of maize stalks below 0.26 resulted in cocoa pod husk as the optimal crop residue. Ayamga et al. [57] suggested that for low-case residue scenarios, a recoverability ratio of 0.1 should be considered. The outcome of the sensitivity analysis remains unchanged when a recoverability ratio of 0.1 is used for the residues of interest. Contrary to the observations in the individual crop residues analysis, the use of a recoverability ratio of 0.1 for cassava peels did not result in a different outcome for the categories that had cassava peels (Ca and Cc). Similarly, there were no significant changes in the rankings of the various feedstock blends when a 0.1 recoverability ratio was used for all residue types. The results suggest that the correct prediction of the techniques is dependent on using the correct and realistic recoverability ratio.

RPR is the ratio by weight of residues to the corresponding quantity of product harvested. RPR could differ for the same residue types for various farms and nations due to variations in moisture content at the time of measurement, residue, and crop yield which are all dependent on farm management practices and climatic conditions [55]. An RPR value of 0.34 reported by Kemausuor et al. [4] was used for the base case scenario for cassava peels. A lower recoverability ratio of 0.25 has been reported [58]. Maize stalk was ranked as the optimal crop residues for gasification in Ghana by TOPSIS and COPRAS when the recoverability of ratio of cassava peels of 0.25 was used.

VIKOR ranked maize stalk as the best alternative at a low RPR value of maize stalk of 1.28 [59]. The Spearman's rank correlation between the techniques was observed to decrease with a decrease in RPR values. In the VIKOR method, the value of v of 0.5 was used in the base case scenarios. Its value lies between 0 and 1 [60]. Variation of v affected the ranking of the alternatives particularly when v is 0 or 1 (see Table 6). When v is 1 and 0 the best-ranked crop residues were shea nut shells and cocoa pod husk respectively. In all other cases, maize stalk was ranked as the best alternative. Similar sensitivity results were also observed in the various feedstock categories considered.

**Table 6:** Values of Q_j_ for different values of v.

v	A1	A2	A3	A4	A5	A6	A7	A8	A9	A10	A11	A12	A13
0	0.80	0.47	0.13	0.26	0.42	0.98	0.71	0.75	0.63	1.00	0.76	0.00	0.98
0.2	0.84	0.49	0.14	0.26	0.36	0.86	0.67	0.69	0.60	0.97	0.66	0.08	0.79
0.4	0.88	0.52	0.14	0.27	0.30	0.74	0.63	0.63	0.58	0.94	0.57	0.17	0.59
0.5	0.90	0.53	0.14	0.27	0.27	0.67	0.61	0.60	0.57	0.92	0.52	0.21	0.49
0.6	0.92	0.55	0.14	0.27	0.24	0.61	0.59	0.57	0.56	0.91	0.47	0.25	0.39
0.8	0.96	0.57	0.15	0.28	0.17	0.49	0.55	0.50	0.54	0.88	0.37	0.33	0.20
1	1.00	0.60	0.15	0.28	0.11	0.37	0.51	0.44	0.51	0.85	0.27	0.41	0.00

## 4 Conclusion

Selecting the best from available crop residues for optimal gasification requires a holistic approach involving a multiplicity of factors. In this paper, we have shown how VIKOR, TOPSIS, and COPRAS MCDM methods are used to select crop residues for optimal and sustainable gasification in Ghana, among thirteen crop residues with twelve criteria. AHP was used to assign the weight for each criterion considered. Five scenarios were considered; ranking of individual feedstock, consideration of a combination of the feedstock grouped under four categories Ca, Cb, Cc, and Cd. 255 alternatives were considered under alternatives Ca and Cb and 31 alternatives under Cc and Cd. Ranking of the individual crop residues revealed cassava peels as the best alternatives by TOPSIS and COPRAS while VIKOR ranked maize stalk as the best crop residue for optimal gasification in Ghana. The average ranking of the alternatives from the three MCDM in the order of best to worst is cassava peels, maize stalk, cocoa pod husk, maize cobs, rice straw, shea nut husk, groundnut husk, Palm kernel shells, EFB, rice husk, coconut husk/shells, cashew shells, and shea nut cake. Rankings of the alternatives under the four categories considered revealed that feedstock blends containing four residues or more offer better opportunities for sustainable gasification than individual feedstocks. Statistical analysis shows that there is no significant difference between the rankings of the alternatives by the three MCDM techniques and that there is a very strong, positive correlation between VIKOR, COPRAS, and TOPSIS. The strongest correlation is between VIKOR and TOPSIS with Spearman's rank correlation index of 0.98. The strength of the correlation generally increases with an increase in the number of alternatives. On the contrary opposite effect on the size of alternatives on the spearman's rank correlation was observed between COPRAS and the other two techniques. The analysis was sensitive to the weight of the strategy of group utility (v), recoverability ratio, RPR and annual crop production figures. It is recommended that optimal gasifier design and operational conditions taken into consideration the various feedstocks and their combination as determined from this study must be studied. The gasification of crop residues for optimal energy generation is expected to contribute to sustainable energy generation in Ghana, particularly for mini-grid and off-grid power systems for remote rural agricultural communities and the generation of heat for crop processing. The development of the gasification systems along the identified residue types as presented in this study is expected to contribute to the development of the sector.

## Author contribution statement

Isaac Osei: Conceived and designed the experiments; Performed the experiments; Wrote the paper.

Ahmad Addo: Analyzed and interpreted the data; Contributed reagents, materials, analysis tools or data; Wrote the paper.

Francis Kemausuor: Analyzed and interpreted the data; Wrote the paper.

## Data availability statement

Data included in article/supp. material/referenced in article.

## Additional information

No additional information is available for this paper.

## Declaration of competing interest

The authors declare that they have no known competing financial interests or personal relationships that could have appeared to influence the work reported in this paper.

## Figures and Tables

**Table A1:** Crop Residue Combination under each Category.

Categories	Crop residue types
Category a (Ca)	A1, A2, A3, A4, A5, A8, A9 and A12.
Category b (Cb)	A1, A2, A3, A4, A7, A8, A9 and A12
Category c (Cc)	A3, A4, A5, A6, A12
Category d (Cd)	A1, A2, A10, A11, and A13

**Table A3:** Determination of cost factors for crop residue types

Residue Type	Competition of Residues	Difficulties in transportation	Residue Cluster	Calorific value	Total value
A1	0.014	0.063	0.020	0.165	**0.26**
A2	0.009	0.100	0.190	0.187	**0.49**
A3	0.007	0.090	0.180	0.182	**0.46**
A4	0.104	0.040	0.150	0.201	**0.49**
A5	0.250	0.030	0.080	0.187	**0.55**
A6	0.400	0.016	0.100	0.300	**0.82**
A7	0.105	0.014	0.200	0.210	**0.53**
A8	0.201	0.004	0.140	0.237	**0.58**
A9	0.128	0.050	0.140	0.187	**0.50**
A10	0.250	0.021	0.040	0.292	**0.60**
A11	0.152	0.070	0.060	0.208	**0.49**
A 12	0.032	0.025	0.070	0.183	**0.31**
A13	0.127	0.043	0.040	0.221	**0.43**
	0-lowest competition to 0.4 highest competition	0-not difficult to transport 0.1-difficult to transport	0-residue clustered at one place to 0.2-residue dispersed	0-Lowest heating value 0.3-highest heating value	
Reference	[4]	[[47], [48], [49], [50]]	[4]	(Calorific values as determined in this study (see Table 4))	

**Table A4:** Pairwise comparison matrix for the criteria.

	PS	MC_d_	PA	PC	PV	C	H	O	S	N	Ct	Q
PS	1	3	7	1/7	1/5	1/6	1/4	1/2	7	5	1/5	1/6
MC_d_	1/3	1	4	1/8	1/7	1/7	1/5	1/4	6	2	1/7	1/9
PA	1/7	1/4	1	1/9	1/9	1/9	1/9	1/7	2	1/3	1/9	1/9
PC	7	8	9	1	2	2	3	5	9	9	2	1/3
PV	5	7	9	1/2	1	1/2	1/2	1/2	9	9	1/3	1/5
C	6	7	9	1/2	2	1	2	4	9	8	2	1/4
H	4	5	9	1/3	2	1/2	1	3	9	8	1/3	1/5
O	2	4	7	1/5	2	1/3	1/3	1	9	8	1/3	1/5
S	1/7	1/6	1/2	1/9	1/9	1/9	1/9	1/9	1	1/2	1/9	1/9
N	1/5	1/2	3	1/9	1/9	1/8	1/8	1/8	2	1	1/9	1/9
Ct	5	7	9	1/2	3	1/2	3	3	9	9	1	2
Q	6	9	9	3	5	4	5	5	9	9	1/2	1

**Table A5:** Crop residue constituent for the alternatives under Category Ca and Cb.

Alternative (Ca)	Residue types	Alternatives (Cb)	Residue types
Ca155	A1,A2,A3,A4,A5	Cb155	A1,A2,A3,A4,A8
Ca161	A1,A2,A3,A5,A12	Cb161	A1,A2,A3,A8,A7
Ca176	A1,A3,A4,A5,A9	Cb176	A1,A3,A4,A8,A12
Ca177	A1,A3,A4,A5,A12	Cb177	A1,A3,A4,A8,A7
Ca191	A2,A3,A4,A5,A9	Cb191	A2,A3,A4,A8,A12
Ca192	A2,A3,A4,A5,A12	Cb192	A1,A3,A4,A12,A7
Ca213	A1,A2,A3,A4,A5,A12	Cb212	A1,A2,A3,A4,A8,A12
Ca228	A1,A3,A4,A5,A9,A12	Cb213	A1,A2,A3,A4,A8,A7
Ca234	A2,A3,A4,A5,A9,A12	Cb228	A1,A3,A4,A8,A12,A7
Ca239	A1,A2,A3,A4,A5,A8,A9	Cb234	A2,A3,A4,A8,A12,A7
Ca240	A1,A2,A3,A4,A5,A8,A12	Cb239	A1,A2,A3,A4,A8,A9,A12
Ca241	A1,A2,A3,A4,A5,A9,A12	Cb240	A1,A2,A3,A4,A8,A9,A7
Ca243	A1,A2,A3,A5,A8,A9,A12	Cb241	A1,A2,A3,A4,A8,A12,A7
Ca244	A1,A2,A4,A5,A8,A9,A12	Cb243	A1,A2,A3,A8,A9,A12,A7
Ca245	A1,A3,A4,A5,A8,A9,A12	Cb244	A1,A2,A4,A8,A9,A12,A7
Ca246	A2,A3,A4,A5,A8,A9,A12	Cb245	A1,A3,A4,A8,A9,A12,A7
Ca247	A1,A2,A3,A4,A5,A8,A9,A12	Cb246	A2,A3,A4,A8,A9,A12,A7
		Cb247	A1,A2,A3,A4,A8,A9,A12,A7

**Table A6:** Crop residue constituent for the alternatives under Category Cc and Cd.

Alternative (Cc)	Residue types	Alternative (Cd)	Residue types
Cc1	A3,A4	Cd1	A1,A2
Cc2	A3,A5	Cd2	A1,A10
Cc3	A3,A6	Cd3	A1,A11
Cc4	A3,A12	Cd4	A1,A13
Cc5	A4,A5	Cd5	A2,A10
Cc6	A4,A6	Cd6	A2,A11
Cc7	A4,A12	Cd7	A2,A13
Cc8	A5,A6	Cd8	A10,A11
Cc9	A5,A12	Cd9	A10,A13
Cc10	A6,A12	Cd10	A11,A13
Cc11	A3,A4,A5	Cd11	A1,A2,A10
Cc12	A3,A4,A6	Cd12	A1,A2,A11
Cc13	A3,A4,A12	Cd13	A1,A2,A13
Cc14	A3,A5,A6	Cd14	A1,A10,A11
Cc15	A3,A5,A12	Cd15	A1,A10,A13
Cc16	A3,A6,A12	Cd16	A1,A11,A13
Cc17	A4,A5,A6	Cd17	A2,A10,A11
Cc18	A4,A5,A12	Cd18	A2,A10,A13
Cc19	A4,A6,A12	Cd19	A2,A11,A13
Cc20	A5,A6,A12	Cd20	A10,A11,A13
Cc21	A3,A4,A5,A6	Cd21	A1,A2,A10,A11
Cc22	A3,A4,A5,A12	Cd22	A1,A2,A10,A13
Cc23	A3,A4,A6,A12	Cd23	A1,A2,A11,A13
Cc24	A3,A5,A6,A12	Cd24	A1,A10,A11,A13
Cc25	A4,A5,A6,A12	Cd25	A2,A10,A11,A13
Cc26	A3,A4,A5,A6,A12	Cd26	A1,A2,A10,A11,A13

**Table A2:** Estimation of Dry Technical Residue quantities for each residue type

Residue Type				Crop production figures (10^3 tonnes)														
	RPR	$\eta_{reci}$	MC	2009	2010	2011	2012	2013	2014	2015	2016	2017	2018	C (10^3 Tonnes)	Q (10^3 Tonnes)	Lower bound value of Q (10^3 tonnes)	Upper bound value of Q (10^3 tonnes)	Reference for RPR, RR and moisture content
A1	0.23	0.8	13	391	492	463	481	570	604	641	688	722	769	582.12	**93.17**	62.58	123.15	[4]
A2	3.05	0.35	16	391	492	463	481	570	604	641	687	722	769	582.12	**525.09**	352.70	694.03	[4]
A3	1.37	0.35	15	1620	1872	1683	1950	1764	1769	1692	1722	2011	2306	1838.95	**749.33**	660.12	939.80	[4]
A4	0.25	0.8	8	1620	1872	1683	1950	1764	1769	1692	1722	2011	2306	1838.95	**338.33**	298.05	424.33	[4]
A5	0.34	0.2	20	12,231	13,504	14,240	14,547	15,990	16,524	17,213	17,798	19,009	20,846	16,190	**880.75**	665.37	1134.02	[4]
A6	2.1	0.1	7	27	33	36	40	42	50	50	78	90	73	51.87	**10.18**	5.30	17.67	[43,61]
A7	0.54	0.8	12	274	292	344	345	366	380.38	380	380	383	395	354.04	**135.13**	104.50	150.72	[4]
A8	0.065	0.8	6	2104	2004	2126	2196	2334	2451	2530	2443	2465	2604	2325.75	**113.68**	97.97	127.30	[4]
A9	0.23	0.8	60	2104	2004	2126	2196	2334	2451	2530	2443	2465	2604	2325.75	**171.17**	147.52	191.68	[4]
A10	0.2	0.5	7	39	37	32.655	33	34	34	34	34.	34	33	34.50	**3.21**	3.04	3.61	[62,63]
A11	0.35	0.8	14	485	531	465	475	409	427	417	426	434	521	458.96	**110.75**	98.69	128.13	[4]
A 12	1	0.8	15	711	632	700	879	835	859	859	859	894	948	817.49	**555.89**	429.79	644.39	[4]
A13	0.6	0.5	2	39	37	33	33	34	34	34	34	34	33	34.45	**10.15**	9.61	11.42	[62,63]
